# Supplementary material for: Improved quality metrics for association and reproducibility in chromatin accessibility data using mutual information
Source: BMC Bioinformatics. 2023 Nov 22;24:441. doi: 10.1186/s12859-023-05553-0 (PMC10664258; doi:10.1186/s12859-023-05553-0)
Supplement: Supplementary file 1 — Additional file 1: Figure S1. Boxplots displaying the area under the curve (y-axis) across statistics (x-axis) with co-zeros retained and removed from analysis (blue andorange boxes, respectively). [file 12859_2023_5553_MOESM1_ESM.pdf]

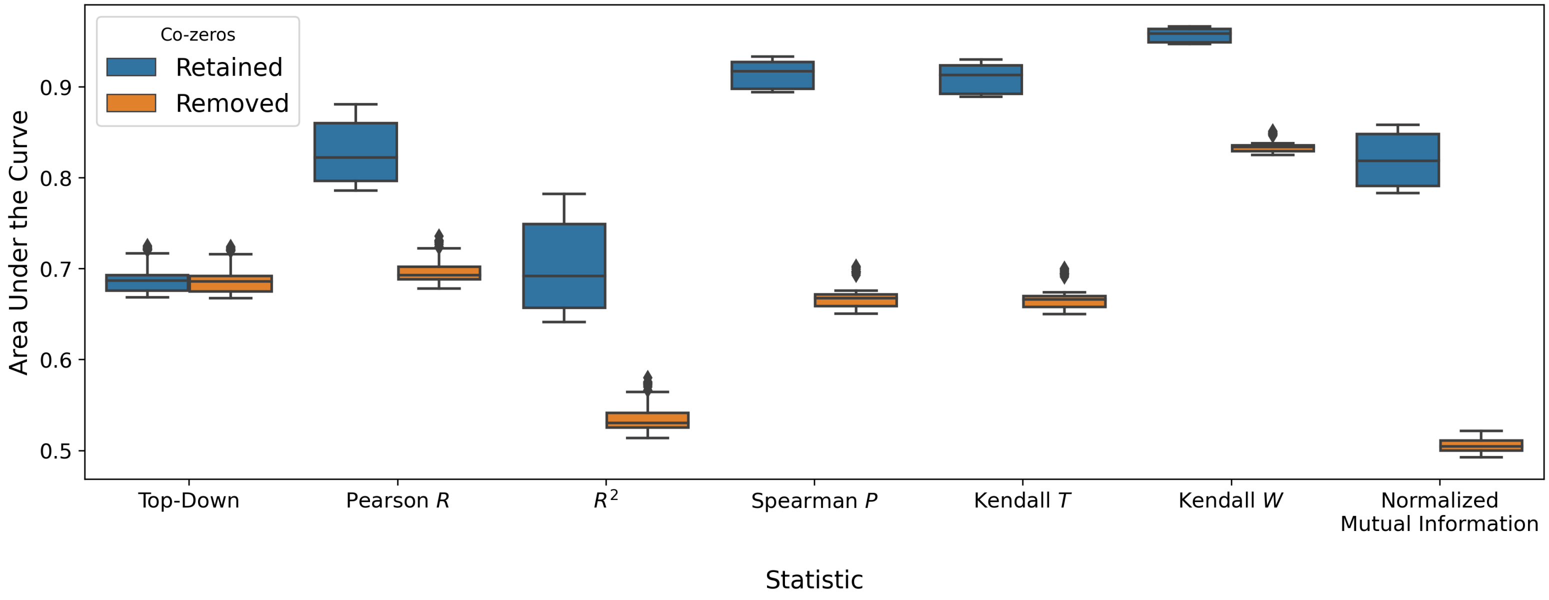

Figure S1: Boxplots displaying the area under the curve (y-axis) across statistics (x-axis) with co-zeros retained and removed from analysis (blue and orange boxes, respectively).
