# Supplementary material for: Improved quality metrics for association and reproducibility in chromatin accessibility data using mutual information
Source: BMC Bioinformatics. 2023 Nov 22;24:441. doi: 10.1186/s12859-023-05553-0 (PMC10664258; doi:10.1186/s12859-023-05553-0)
Supplement: Supplementary file 2 — Additional file 2: Figure S2. Bi-variate plot of WFpkm counts (across 10 kb genomic bins) between replicates of real, A549ATAC-seq experiments. Dark red to blue colors and marker size designate the density (log10 (WFpkmcounts)) of counts between replicates. Co-zero values appear as an orange dot in lower left corner. A dashedgrey line represents a one-to-one relationship between the two replicates. [file 12859_2023_5553_MOESM2_ESM.pdf]

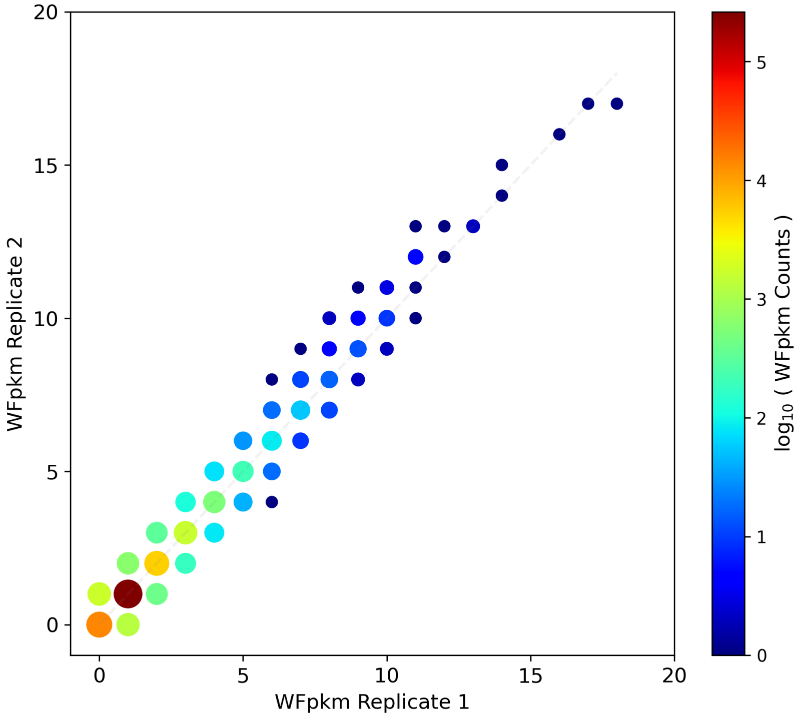

Figure S2: Bi-variate plot of WFpkm counts (across 10 kb genomic bins) between replicates of real, A549 ATAC-seq experiments. Dark red to blue colors and marker size designate the density (log10 (WFpkm counts)) of counts between replicates. Co-zero values appear as an orange dot in lower left corner. A dashed grey line represents a one-to-one relationship between the two replicates.
