# Supplementary material for: Improved quality metrics for association and reproducibility in chromatin accessibility data using mutual information
Source: BMC Bioinformatics. 2023 Nov 22;24:441. doi: 10.1186/s12859-023-05553-0 (PMC10664258; doi:10.1186/s12859-023-05553-0)
Supplement: Supplementary file 3 — Additional file 3: Figure S3. The percent of co-zero values in bi-variate WFpkm distributions between real ATAC-seq experiments.Sample names are annotated along the x- and y-axis. [file 12859_2023_5553_MOESM3_ESM.pdf]

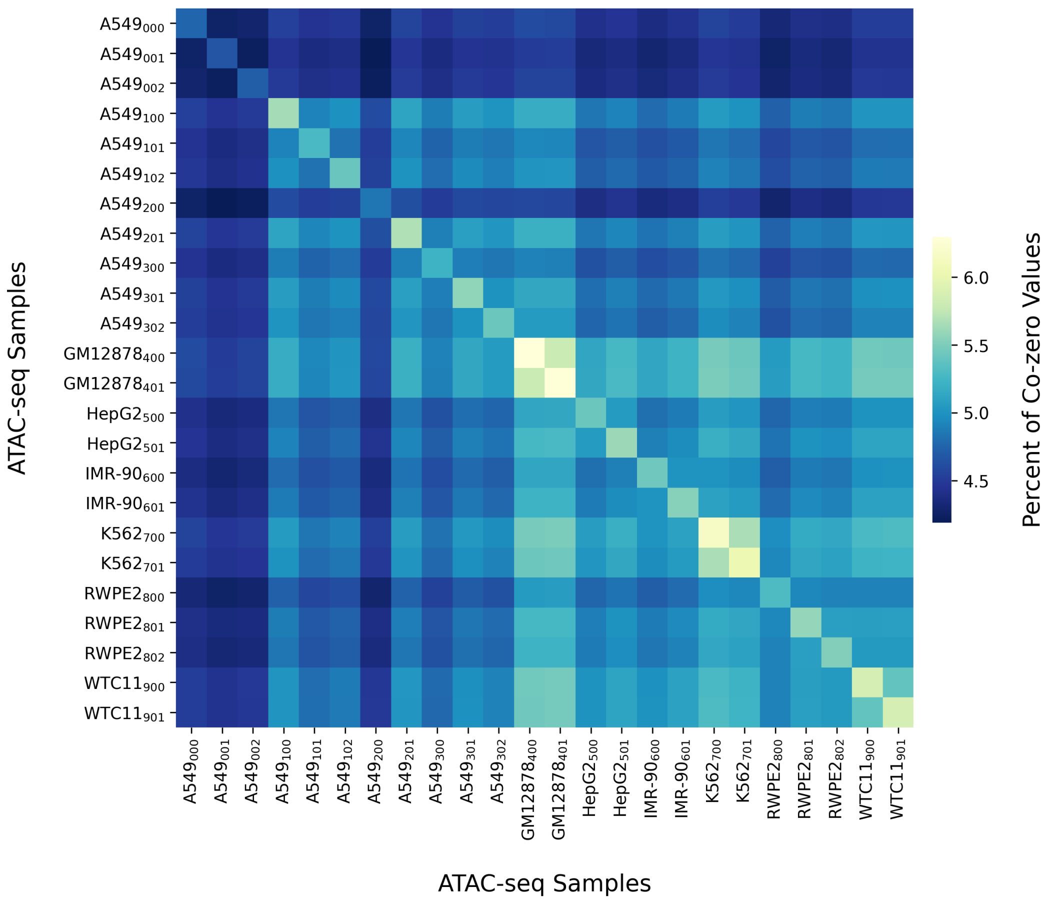

Figure S3: The percent of co-zero values in bi-variate WFpkm distributions between real ATAC-seq experiments. Sample names are annotated along the x- and y-axis.
