# Supplementary material for: Improved quality metrics for association and reproducibility in chromatin accessibility data using mutual information
Source: BMC Bioinformatics. 2023 Nov 22;24:441. doi: 10.1186/s12859-023-05553-0 (PMC10664258; doi:10.1186/s12859-023-05553-0)
Supplement: Supplementary file 4 — Additional file 4: Figure S4. Correlation and association values (y-axis) as a function of percentage of shared peaks betweensynthetic replicates (x-axis). Red and grey curves depict the mean and 95% CI (respectively) values acrosssimulations. A grey, dashed line marks a one-to-one relationship between the x- and y-axis. Left and rightcolumns display change in values as a function of removing co-zeros. Results are from simulations with 50%paired reads within selected peaks removed. [file 12859_2023_5553_MOESM4_ESM.pdf]

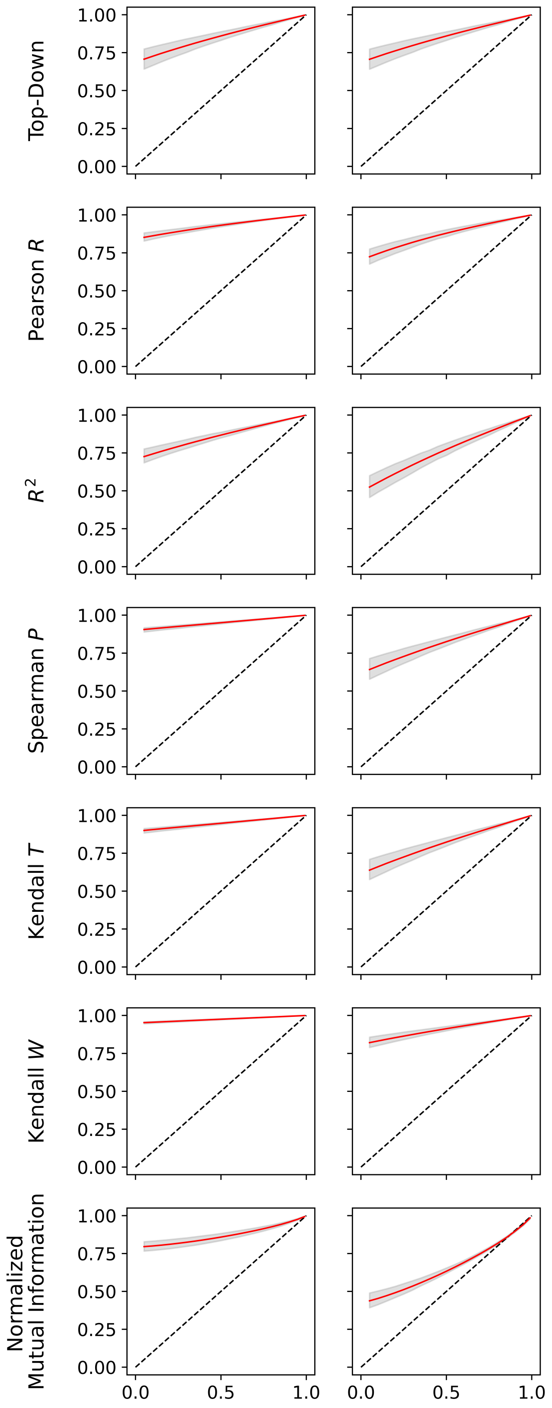

Figure S4: Correlation and association values (y-axis) as a function of percentage of shared peaks between synthetic replicates (x-axis). Red and grey curves depict the mean and 95% CI (respectively) values across simulations. A grey, dashed line marks a one-to-one relationship between the x- and y-axis. Left and right columns display change in values as a function of removing co-zeros. Results are from simulations with 50% paired reads within selected peaks removed.
