# Supplementary material for: Improved quality metrics for association and reproducibility in chromatin accessibility data using mutual information
Source: BMC Bioinformatics. 2023 Nov 22;24:441. doi: 10.1186/s12859-023-05553-0 (PMC10664258; doi:10.1186/s12859-023-05553-0)
Supplement: Supplementary file 6 — Additional file 6: Figure S6. Correlation and association statistics across epigenomic experiments. For samples from AATAC-seq and ChIP-seq (assays for B H3K27ac and C H3K4me3 modifications) experiments, the Spearman’sρ, Pearson’s R, R2 coefficient, and normalized mutual information (x-axis of columns left to right, respectively) were calculated on WFpkm counts between replicates, with (blue) and without co-zeros (orange). [file 12859_2023_5553_MOESM6_ESM.pdf]

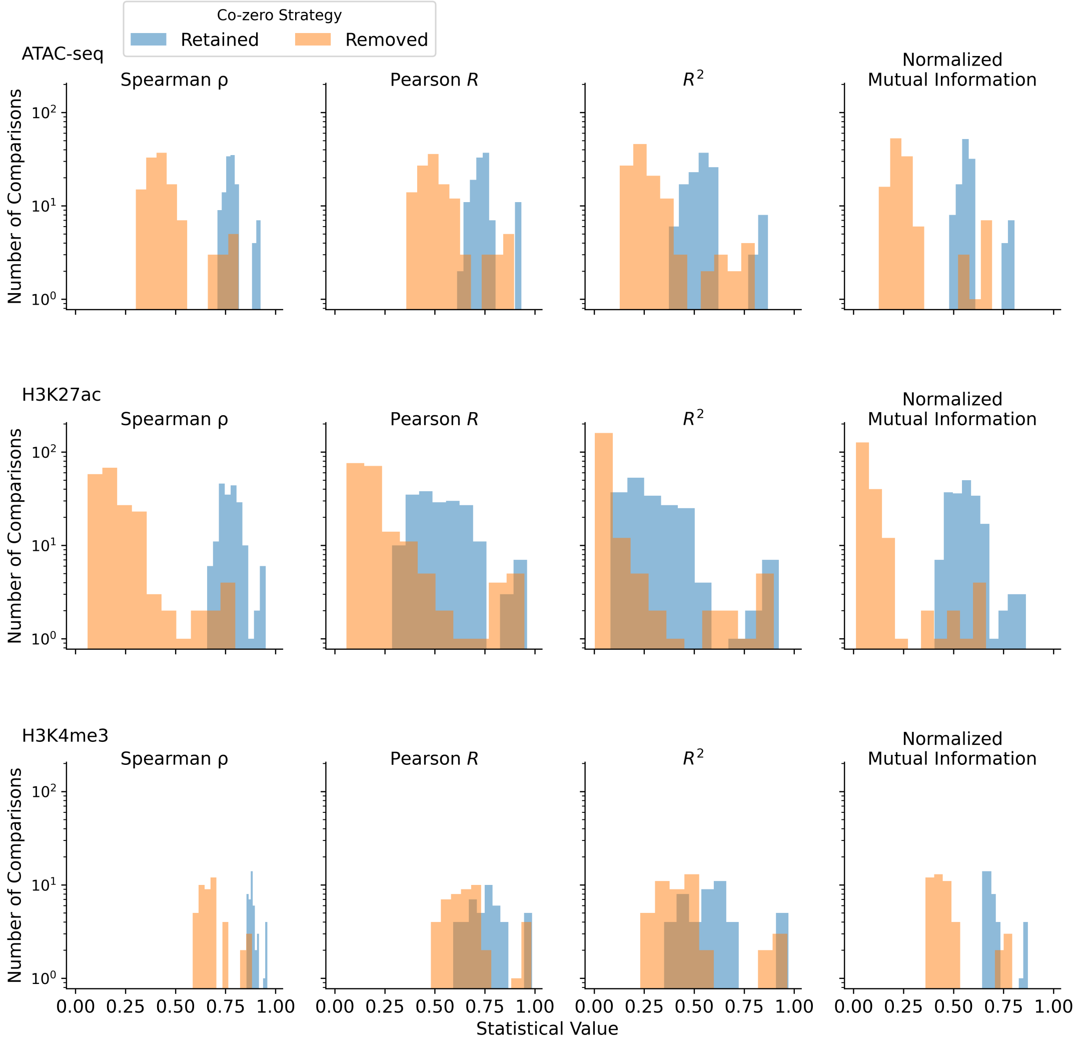

Figure S6: Correlation and association statistics across epigenomic experiments. For samples from (**A**) ATAC-seq and ChIP-seq (assays for (**B**) H3K27ac and (**C**) H3K4me3 modifications) experiments, the Spearman's ρ, Pearson's R, R^2 coefficient, and normalized mutual information (x-axis of columns left to right, respectively) were calculated on WFpkm counts between replicates, with (blue) and without co-zeros (orange).
