# Supplementary material for: Improved quality metrics for association and reproducibility in chromatin accessibility data using mutual information
Source: BMC Bioinformatics. 2023 Nov 22;24:441. doi: 10.1186/s12859-023-05553-0 (PMC10664258; doi:10.1186/s12859-023-05553-0)
Supplement: Supplementary file 7 — Additional file 7: Figure S7. The coefficient of determination (R2) versus the normalized mutual information (y- and x-axis,respectively) calculated on binned counts of WFpkm between ATAC-seq experiments. Blue triangles, orangeXs, and green circles mark comparisons between independent experiments, between independent experimentsusing the same cell line, or true experimental replicates, respectively. [file 12859_2023_5553_MOESM7_ESM.pdf]

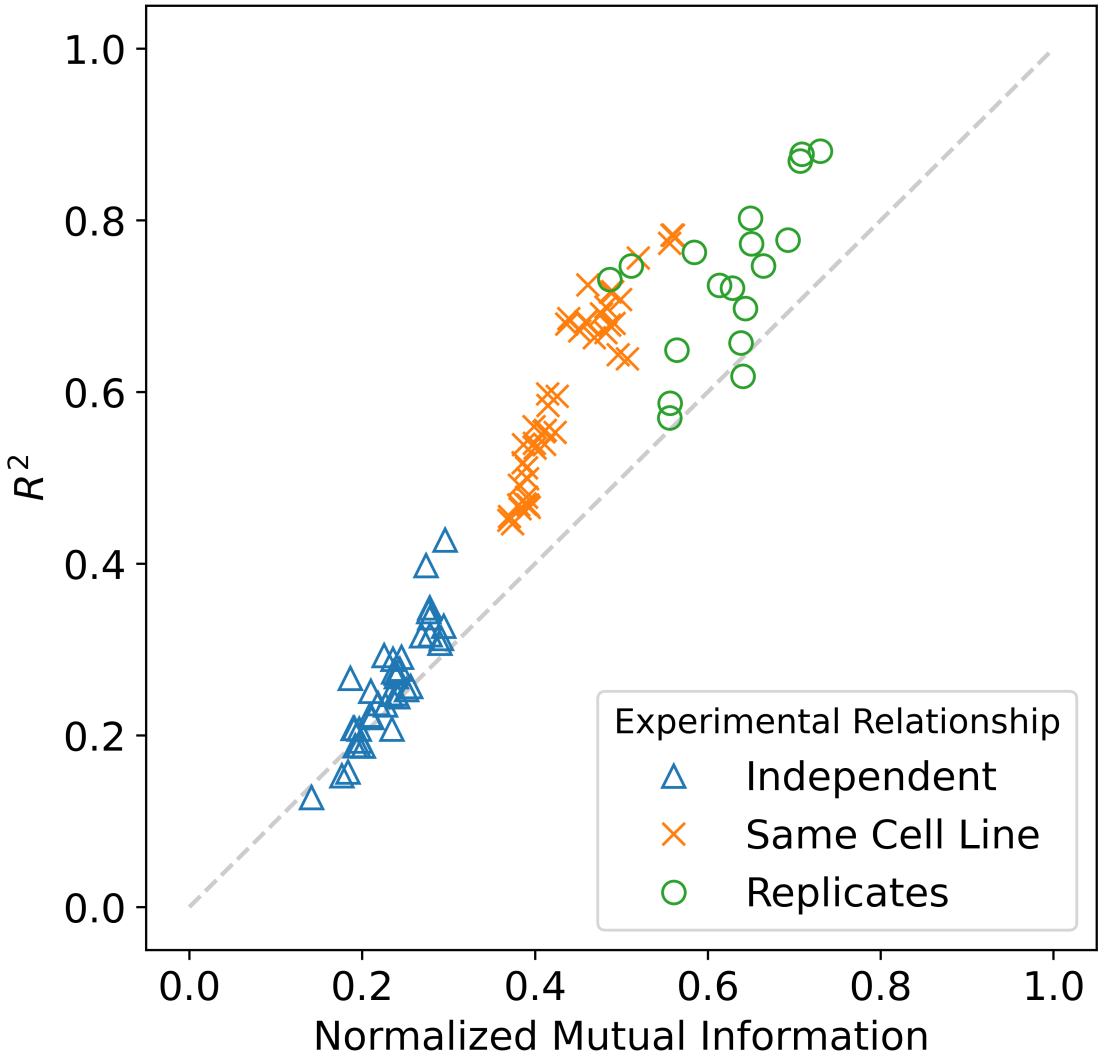

Figure S7: The coefficient of determination (R^2) versus the normalized mutual information (y- and x-axis, respectively) calculated on binned counts of WFpkm between ATAC-seq experiments. Blue triangles, orange Xs, and green circles mark comparisons between independent experiments, between independent experiments using the same cell line, or true experimental replicates, respectively.
