# Supplementary material for: Improved quality metrics for association and reproducibility in chromatin accessibility data using mutual information
Source: BMC Bioinformatics. 2023 Nov 22;24:441. doi: 10.1186/s12859-023-05553-0 (PMC10664258; doi:10.1186/s12859-023-05553-0)
Supplement: Supplementary file 8 — Additional file 8: Figure S8. The f1-scores, recall, and precision of the random forest model with ten-fold, stratified cross validation. Blue, orange, and green colorsdenote experimental relationship class. [file 12859_2023_5553_MOESM8_ESM.pdf]

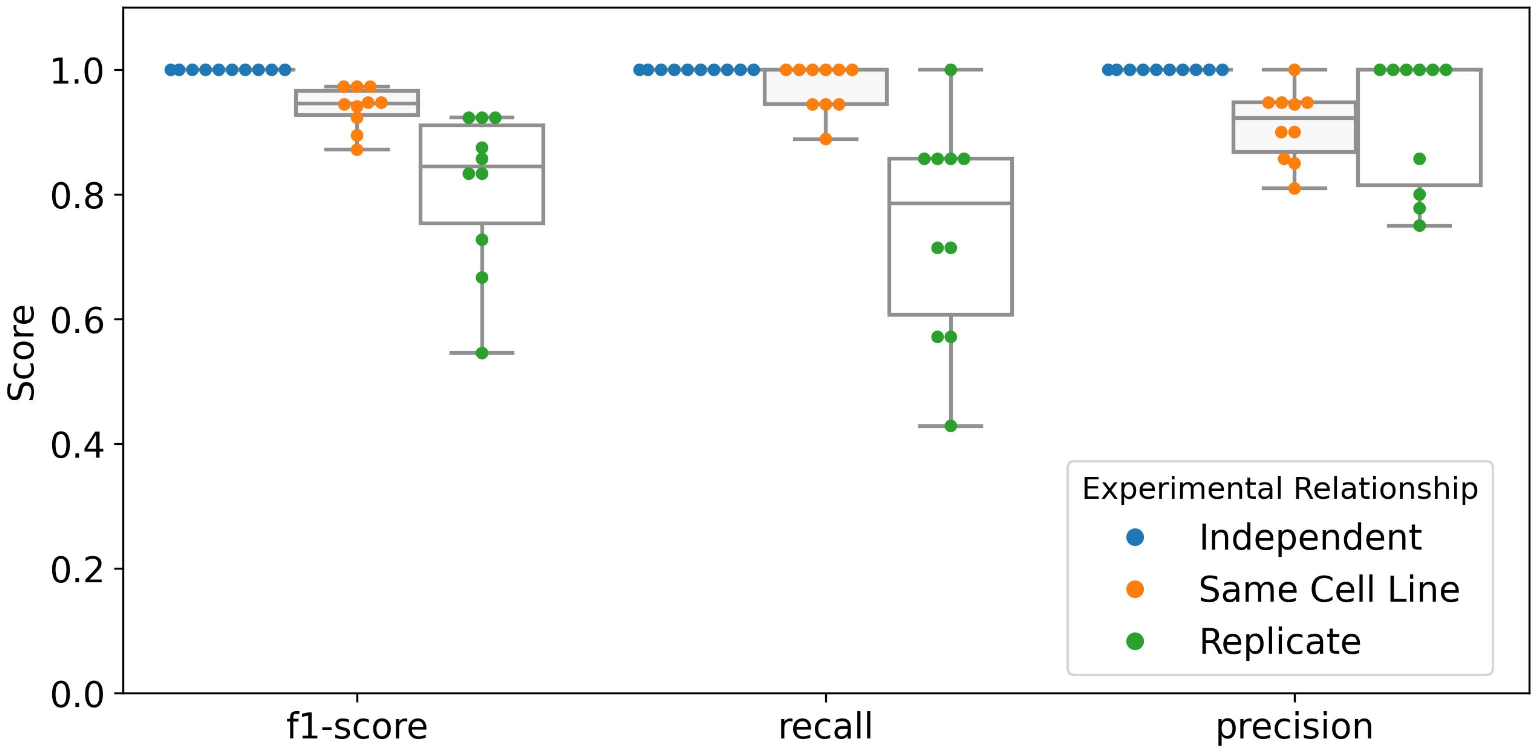

Figure S8: The f1-scores, recall, and precision of the random forest model with ten-fold, stratified cross validation. Blue, orange, and green colors denote experimental relationship class.
