# Supplementary material for: Improved quality metrics for association and reproducibility in chromatin accessibility data using mutual information
Source: BMC Bioinformatics. 2023 Nov 22;24:441. doi: 10.1186/s12859-023-05553-0 (PMC10664258; doi:10.1186/s12859-023-05553-0)
Supplement: Supplementary file 9 — Additional file 9: Table S1. Read counts of ATAC-seq experiments. [file 12859_2023_5553_MOESM9_ESM.pdf]

.

Table S1: Read Counts of ATAC-seq Experiments

Sample Title	Cell Line	Total Reads	Mapped Reads	Not Used	mtDNA	Duplicates	Un-mapped	Low Quality Reads	Replicate Name	Source
A549$_{000}$	A549	341325836	259029456	21246814	12009324	46948944	408486	1682812	ENCLB404SKN	ENCSR032RGS
A549$_{001}$	A549	442074976	329679445	27536117	15475857	66338506	506856	2538195	ENCLB605LCC	ENCSR032RGS
A549$_{002}$	A549	277970512	211291691	18456829	11170486	35323112	343051	1385343	ENCLB817BKI	ENCSR032RGS
A549$_{100}$	A549	65405524	23987725	2973344	33653170	3093813	48906	1648566	2501_001	This study
A549$_{101}$	A549	84816540	22605005	2595465	55231224	2481489	32350	1871007	2501_002	This study
A549$_{102}$	A549	64084756	17618743	2122339	40809830	1979951	67472	1486421	2501_003	This study
A549$_{200}$	A549	133625408	35069198	4386111	86279921	4418826	31955	3439397	2501_007	This study
A549$_{201}$	A549	69273610	15377297	1834370	48556785	1780806	27699	1696653	2501_008	This study
A549$_{300}$	A549	86963986	42567716	4788405	31620108	5703777	121028	2162952	2501_018	This study
A549$_{301}$	A549	84297712	28744542	3582876	44737775	5400961	118620	1712938	2501_019	This study
A549$_{302}$	A549	97877188	35836016	4491769	49816243	5663997	42546	2026617	2501_020	This study
GM12878$_{400}$	GM12878	76479882	46889870	4260513	11245729	12635046	252057	1196667	ENCLB584REF	ENCSR095QNB
GM12878$_{401}$	GM12878	69456510	49588811	4319318	7334534	6740176	186878	1286793	ENCLB907YRF	ENCSR095QNB
HepG2$_{500}$	HepG2	76077306	48113686	6037783	8348893	11668633	235020	1673291	ENCLB074EQT	ENCSR042AWH
HepG2$_{501}$	HepG2	88838406	48246610	6580203	19207768	12021756	605060	2177009	ENCLB324GIU	ENCSR042AWH
IMR-90$_{600}$	IMR-90	84117916	47543633	11830808	8448694	9559287	5990188	745306	ENCLB432QLN	ENCSR200OML
IMR-90$_{601}$	IMR-90	95034796	61359070	6202820	14378540	10233756	1872742	987868	ENCLB937FOM	ENCSR200OML
K562$_{700}$	K562	78745422	48217636	6777147	10759718	10705486	91659	2193776	ENCLB758GEG	ENCSR483RKN
K562$_{701}$	K562	83982064	52270533	6752478	10447009	12175330	162811	2173903	ENCLB918NXF	ENCSR483RKN
RWPE2$_{800}$	RWPE2	67263926	55152003	6718663	753542	2685741	286519	1667458	ENCLB293SLX	ENCSR080SNF
RWPE2$_{801}$	RWPE2	53441754	43166947	5244472	1277887	2088775	323207	1340466	ENCLB734LAL	ENCSR080SNF
RWPE2$_{802}$	RWPE2	60212304	48162285	5946340	1888964	2288274	331284	1595157	ENCLB984XHJ	ENCSR080SNF
WTC11$_{900}$	WTC11	115952320	74558506	7218516	7595855	21538753	4422396	618294	ENCLB621FEI	ENCSR541KFY
WTC11$_{901}$	WTC11	127343084	79335328	7889715	9553155	24757738	5028262	778886	ENCLB715JYV	ENCSR541KFY
