# Supplementary material for: Improved quality metrics for association and reproducibility in chromatin accessibility data using mutual information
Source: BMC Bioinformatics. 2023 Nov 22;24:441. doi: 10.1186/s12859-023-05553-0 (PMC10664258; doi:10.1186/s12859-023-05553-0)
Supplement: Supplementary file 10 — Additional file 10: Table S2. Fragment counts of ChIP-seq experiments from the ENCODE project. [file 12859_2023_5553_MOESM10_ESM.pdf]

.

Table S2: Fragment Counts of ChIP-seq Experiments from the ENCODE Project

ENCODE Accession	Assay	Cell Line	Max WFpkm (10 kb bins)	Filtered Fragments	Total Fragments
ENCFF296OMI	H3K27ac	22Rv1	20	38716637	42868881
ENCFF434UTC	H3K27ac	22Rv1	25	22880850	25208977
ENCFF474LGV	H3K27ac	C4-2B	13	40421083	45275154
ENCFF593VYY	H3K27ac	C4-2B	23	24832367	27534380
ENCFF363VMR	H3K27ac	HL-60	30	77226537	86957892
ENCFF896NMH	H3K27ac	HL-60	38	86535096	97330610
ENCFF581PPG	H3K27ac	NCI-H929	40	78893815	88952938
ENCFF627SMP	H3K27ac	NCI-H929	45	78892515	88237795
ENCFF408GVF	H3K27ac	RWPE1	40	80806746	87845230
ENCFF806MCZ	H3K27ac	RWPE1	41	77080881	84044868
ENCFF191AEE	H3K27ac	RWPE2	40	52686940	56819777
ENCFF800SRX	H3K27ac	RWPE2	46	34244335	36427250
ENCFF858VHX	H3K27ac	SJCRH30	20	70130048	77261532
ENCFF936CKN	H3K27ac	SJCRH30	14	84535928	94990790
ENCFF264EZF	H3K27ac	SJSA1	26	68560573	77365636
ENCFF364AIR	H3K27ac	SJSA1	29	63590794	71144919
ENCFF613MAH	H3K27ac	VCaP	26	28002993	30184213
ENCFF967YSF	H3K27ac	VCaP	16	52998296	57727449
ENCFF417QIX	H3K27ac	WERI-Rb-1	8	69869255	77981097
ENCFF635LGT	H3K27ac	WERI-Rb-1	9	70912815	79055950
ENCFF683RST	H3K4me3	HAP-1	13	78112300	86716019
ENCFF941CSK	H3K4me3	HAP-1	11	90845864	100953140
ENCFF289JFI	H3K4me3	MG63	31	64534217	70898108
ENCFF778ZUK	H3K4me3	MG63	32	77422930	84905617
ENCFF639EED	H3K4me3	RWPE2	10	69807038	79648038
ENCFF696EBU	H3K4me3	RWPE2	11	65118423	75286399
ENCFF249YKT	H3K4me3	SJCRH30	55	77134376	83941981
ENCFF409CAG	H3K4me3	SJCRH30	51	79654231	86985228
ENCFF759EUU	H3K4me3	SJSA1	85	66510060	74121515
ENCFF942YVE	H3K4me3	SJSA1	82	68668188	76886308
