# Supplementary material for: Improved quality metrics for association and reproducibility in chromatin accessibility data using mutual information
Source: BMC Bioinformatics. 2023 Nov 22;24:441. doi: 10.1186/s12859-023-05553-0 (PMC10664258; doi:10.1186/s12859-023-05553-0)
Supplement: Supplementary file 11 — Additional file 11: Table S3. Difference of mean correlation and association values between replicates and non-replicates. [file 12859_2023_5553_MOESM11_ESM.pdf]

.

Table S3: Difference of Mean Correlation and Association values between Replicates and Non-replicates

Assay	Statistic	Differnce in Means (with Co-zeros)	Difference in Means (without Co-zeros)
ATAC-seq	Normalized Mutual Information	0.22	0.4
ATAC-seq	Pearson R	0.19	0.34
ATAC-seq	R^2	0.32	0.45
ATAC-seq	Spearman ρ	0.14	0.34
H3K27ac	Normalized Mutual Information	0.23	0.47
H3K27ac	Pearson R	0.41	0.69
H3K27ac	R^2	0.58	0.71
H3K27ac	Spearman ρ	0.15	0.49
H3K4me3	Normalized Mutual Information	0.18	0.32
H3K4me3	Pearson R	0.23	0.32
H3K4me3	R^2	0.39	0.5
H3K4me3	Spearman ρ	0.07	0.2
